# Prevention and Treatment of Sarcopenia: Multidisciplinary Approaches in Clinical Practice

**DOI:** 10.3390/nu15092163

**Published:** 2023-04-30

**Authors:** Yoshihiro Yoshimura

**Affiliations:** Center for Sarcopenia and Malnutrition Research, Kumamoto Rehabilitation Hospital, 760 Magate, Kikuchi-County, Kumamoto 869-1106, Japan; hanley.belfus@gmail.com; Tel.: +81-96-232-3111; Fax: +81-96-232-3119

Sarcopenia is a common clinical problem in older people and often leads to severe adverse outcomes. The growing interest in sarcopenia has highlighted the need to understand more about its management. The preservation or improvement of physical function and independent living are vital in frail older adults, and sarcopenia is a major contributor to physical frailty. This Special Issue updates our knowledge on the prevention and treatment of sarcopenia and includes clinically and academically intriguing and practical research findings on nutrition, exercise, and drugs that should be included in interventions.

Nistor-Cseppento CD et al. discuss the potential benefits of diet therapy and probiotics in treating sarcopenia induced by prolonged immobilization caused by the COVID-19 pandemic [1]. The authors suggest that increasing protein intake and consuming specific probiotics that promote muscle anabolism, along with adequate physical training, can help improve skeletal muscle mass index (SMI) in patients with sarcopenia. However, the authors acknowledge that more research is needed to fully understand the effectiveness of these treatments and to elucidate the mechanisms through which some probiotics can influence sarcopenia.

Ohtsubo T et al. investigate the association between objectively measured physical activity and functional improvement in hospitalized patients with sarcopenia [2]. The study was performed according to the guidelines of the Declaration of Helsinki and approved by the Ethics Committee of Konan Women’s University. Informed consent was obtained from all subjects involved in the study. The results suggest that higher levels of physical activity are associated with greater improvements in physical function in patients with sarcopenia during hospitalized rehabilitation. This information can be used to develop effective interventions for these patients.

Exercise is one of the most important interventions to combat sarcopenia. Exercise therapy includes resistance training, aerobic exercise, and balance exercises; however, there has been a lack of evidence on the specific types of exercise to counteract sarcopenia. Yoshimura et al. conducted a cohort study of post-stroke sarcopenia patients and showed that chair stand exercise was effective in improving sarcopenia [3]. Chair stand exercise does not require special equipment and can be performed safely anywhere, and the movement of the exercise itself is an important component of the activities of daily living. Furthermore, chair stand exercise has been reported to be associated with improvement in dysphagia after stroke [4], and these findings suggest that it is a useful and practical exercise to combat sarcopenia when combined with appropriate nutritional therapy [5].

Trends in sarcopenia intervention research would also inform the next generation of research. In a review article by Wu L et al., a bibliometric analysis of nutritional research on sarcopenia was conducted to provide insight into the current state of research on sarcopenia and to identify potential areas for future research [6]. The study found that research on nutrition in the treatment of sarcopenia has developed rapidly over the past decade, with several highly regarded publications. The authors suggest that conducting a bibliometric study related to nutrition and sarcopenia would be significant as a reference for the next phase of development in this area.

Evidence on polypharmacy in patients with sarcopenia is lacking. Polypharmacy is associated with poor patient outcome. Polypharmacy is associated with a poor prognosis in elderly patients. Ongoing research on this topic in the field of rehabilitation medicine shows that patients requiring rehabilitation tend to have more polypharmacy, which is negatively associated with important outcomes such as activities of daily living and discharge destination [7,8,9]. In a cohort study of older patients with sarcopenia undergoing post-stroke convalescent rehabilitation, Matsumoto et al. showed that polypharmacy was associated with dysphagia and undernutrition [10]. These findings suggest that medication management as well as nutrition and exercise are important for sarcopenia prevention and treatment interventions.

Deprescribing from polypharmacy is associated with better outcomes among sarcopenic patients. Deprescribing is the process of tapering or stopping drugs, aimed at minimizing polypharmacy and improving patient outcomes [11], while a review of deprescribing interventional studies among older people reported that very few reported clinical or patient-reported outcomes [12]. A study found that deprescribing from polypharmacy on admission is positively associated with functional status at discharge and home discharge in older patients with sarcopenia after stroke [13]. Another study found that deprescribing leads to improved energy intake among hospitalized older sarcopenic adults with polypharmacy after stroke [14].

Finally, Yoshimura et al. reported on the validity of diagnostic methods for the new concept of sarcopenic obesity [15]. The study aimed to examine the prevalence of sarcopenic obesity as diagnosed by the criteria recently proposed by the European Society for Clinical Nutrition and Metabolism (ESPEN) and the European Association for the Study of Obesity (EASO), and its association with outcomes among patients after stroke, showing that the prevalence of sarcopenic obesity diagnosed by the ESPEN/EASO-defined criteria was as low as 4.5% among Japanese patients after stroke. Furthermore, sarcopenic obesity was negatively associated with improvements in activities of daily living and dysphagia in the study patients.

The purpose of this Special Issue was to update knowledge about the prevention and treatment of sarcopenia, including nutrition, exercise, drug interventions, and other potential interventions. These include probiotics, physical activity, chair stand exercise, polypharmacy, and its discontinuation (deprescribing), all of which are sarcopenia countermeasures that can be implemented relatively easily in clinical practice in real-world settings. The studies discussed in this Special Issue, as well as those published in the wider scientific literature, are important for the continued implementation to combat sarcopenia in today’s aging society. Future, high-quality intervention studies are expected to be continuously conducted to further deepen our knowledge in this area.
